# THE GERMAN DOSE RATE MONITORING NETWORK AND IMPLEMENTED DATA HARMONIZATION TECHNIQUES

**DOI:** 10.1093/rpd/ncy154

**Published:** 2018-01-10

**Authors:** U Stöhlker, M Bleher, H Doll, H Dombrowski, W Harms, I Hellmann, R Luff, B Prommer, S Seifert, F Weiler

**Affiliations:** 1 Bundesamt für Strahlenschutz (Federal Office for Radiation Protection), Willy-Brandt-Straße 5, Salzgitter, Germany; 2 Physikalisch-Technische Bundesanstalt, Bundesallee 100, Braunschweig, Germany

## Abstract

Environmental radiation monitoring networks have been established in Europe and world-wide for the purpose of protecting population and environment against ionizing radiation. Some of these networks had been established during the cold war period and were improved after the Chernobyl accident in 1986. Today, the German Federal Office for Radiation Protection (BfS) operates an early warning network with roughly 1800 ambient dose equivalent rate (ADER) stations equally distributed over the German territory. The hardware and software of all network components are developed in-house allowing the continuous optimization of all relevant components. A probe characterization and quality assurance and control program are in place. Operational and technical aspects of the network and data harmonization techniques are described. The latter allows for calculating of the terrestrial and net ADER combined with uncertainties mainly from site specific effects. Harmonized data are finally used as input to the German emergency management system and the European radiological data exchange platform.

## PURPOSE OF EARLY WARNING NETWORKS AND DATA HARMONIZATION

Consistent and appropriate protective measures must be decided before, during and after radiological emergencies. This requires information about the affected areas, the level of contamination and the actual and future exposure. The German nationwide ADER monitoring network includes ~1800 stationary probes equally distributed over the German territory with a typical distance of 20 km between neighboring probes. The density is increased in the 25 km emergency planning zone around nuclear power plants. These additional probes are installed and operated by complementary networks from federal states. Data are exchanged bilaterally between BfS and the local authorities. In the emergency mode data from all stations can be accessed in almost real-time enabling for the information of the population efficiently and in timely manner.

The monitoring network is part of the German ‘Integrated Measuring and Information System for the Surveillance of Environmental Radioactivity’ (IMIS)^(1)^ and the German National Response Plan, which deals with the consequences of a large-scale radioactive contamination of the environment. In addition, data are transmitted to the European radiological data exchange platform (EURDEP)^(2)^ in order to aggregate a full picture for European decision makers and the public.

Different projects have been related to the harmonization of data from early warning networks in Europe for more than 25 years ago. For example, the Working Group on Environmental Dosimetry (WG 3) of the European Radiation Dosimetry Group (EURADOS) has organized six inter-comparison exercises for the European community of early warning network operators. Focusing on the physical characteristics of the detectors, the results of the inter-comparisons provide basic information needed for data harmonization. In 2004, the AIRDOS project^(3)^ and in 2014 the MetroERM project were initiated. Both projects provided detailed information on European dose rate monitoring networks focusing on probe characteristics, e.g. the sensitivity and other physical properties. In addition, the MetroERM project has included the development of a method for an improved and simplified site characterization.

## ADER DETECTORS

### Site selection and detector installation

Ideally ADER monitoring stations shall be located on extensive flat grassland on natural undisturbed ground. In practice, two rules are applied for the selection of new sites for the installation of detectors. First, detectors have to be installed at the height of 1 m on flat natural ground (grassland) without disturbing buildings in a distance of 20 m around at minimum. Second, taking into account neighboring stations, a specific location has to be selected aiming at an almost homogeneous coverage of the German territory.

New probe locations have to be selected if existing locations have to be dismantled. In such cases pre-selection tools are used to identify potential relevant areas. In a second step, potential measuring sites are visited and specific locations are selected taking into account both, radiological and technical aspects. In a next step, contract negotiations are performed with the owner of the selected premises.

Approximately 160 ADER monitoring stations are installed at meteorological fields of the German Weather Service (Deutscher Wetterdienst, DWD). Apart from this, a wide variety of other partners exists: private companies, schools, water supply companies, airports, owners of private properties and communities. Finally, an expert company is contracted to install the technical equipment.

### Detector design

The ADER detectors are equipped with two Geiger-Müller (GM) tubes for low dose rates (LD) and high dose rates (HD) providing a measurement range from 20 nSv h^−1^ to 5 Sv h^−1^.

Today three different types of detectors are in use: GS05, GS07 (since 2010) and GS08 (since 2015). The GS05 originally included a high voltage generator without on-board microcontroller. Data lines from LD and HD data were directly connected to the data logger which is typically installed in a building in a distance up to 200 m from the probe. To avoid wrong readings, which frequently occurred due to the long distance between the tubes and the data logger, a microcontroller was developed and added to the GS05 detector. In all detector types identical LD and HD GM tubes are used with the main characteristics summarized in Table 1. All detector types contain a high voltage generation circuit. It is electronically stabilized in a temperature range between −20 and +60°C for GS08. All different detector types include a microcontroller directly mounted inside the detector housing for the measurement of the LD and HD GM countings in 10 s intervals. In addition, the high voltage readings at the HD tube, the number of coincidence counts between the LD and HD tube, the temperature at the HD and LD tube, the humidity and the air pressure are recorded. A further parameter is the number of counts in the LD tube following a first detection in a time window of 2 ms, the so-called echo parameter. At background level this number is normally smaller than 2% of the LD tube counts. Higher numbers of echo pulses indicate technical problems of the GM tube mainly due to aged counting gas in the LD tube or vibrations of the tube in case of strong wind. Hence, increased echo counts are flagged for further manual analysis.

**Table 1.** Main characteristics (mean and standard deviation) of the GM tubes used by BfS.

Probe type	Count rate per dose rate (min^−1^ μSv^−1^ h)	Intrinsic background rate (min^−1^)	Dose rate range (μSv h^−1^)	Energy range (keV)	Quantity
LD 70 031 A	962 ± 23	16 ± 5	0.02–1000	35–1250	H*(10)
HD 70 018 A	1.2 ± 0.2	0.20 ± 0.02	20–5 000 000	60–2000	H*(10)

The microcontroller in the detector is connected via a 4-wire cable to the data logger and both systems are interfaced using an ASCII data transfer protocol. Hardware and software of the data logger are developed in-house and the data logger is operated using the open source system Linux. Data are transmitted to one of the six network centers either by a fixed wired network, a mobile network or the Internet. For evaluation purposes, at one selected station satellite communication is used as backup for data transmission aiming to extend redundancy at stations close to NPP.

### Detector development, manufacturing and installation

The nano-coated housing of the GM tubes is purchased from specialized manufacturers (Saphymo or Vacutec). The board for the high voltage generation circuit together with the microcontroller is manufactured by another company (Ultratronic), which is also responsible for the assembly of the complete detector system according to the construction plans developed by BfS. The total price for one station is ~4500 €. This includes the prices for the detector, data logger, uninterruptible power supply, batteries and modem as well as the installation costs, the latter with a mean value of 3000 € per station.

The high installation costs are due to the concept of ideal sites. The most important contribution to the installation costs is the trench for the cable connecting detector and data logger. Therefore, some network operators in Europe tend to minimize the overall costs by mounting detectors on a wall or roof instead of 20 m without buildings.

### Detector characterization

About 15 years ago, BfS has established a maintenance center in Neuherberg near Munich for implementation of a dedicated quality assurance program for new and repaired detectors. Main components of the maintenance center are an open field for long-term observations in Munich, a climate chamber, a lead castle and an irradiation facility^(4)^. In addition, BfS operates an open field site for long-term observations at mount Schauinsland near Freiburg (INTERCAL facility). Up to 20 detectors from different countries are operated in parallel at this place, allowing a direct comparison of readings from all probes over long periods of time under variable climate conditions^(5, 6)^.

#### Determination of the intrinsic background

For the determination of the intrinsic background of the LD and HD GM tubes, a method was developed using reference probes which were characterized in the underground laboratories of PTB, UDO resp. UDO II, during different EURADOS inter-comparison experiments^(7)^. The reference probes are used to determine the residual dose rate in a dedicated lead castle. The terrestrial component of the dose rate is almost negligible in the lead castle (wall thickness of 5 cm) and the reading of the secondary cosmic (SCR) component is reduced to ~60%. The residual component in the lead castle is 22 nSv h^−1^ observed by reference probe, which corresponds to a LD count rate of 27 min^−1^ (counts per minute).

For the determination of the intrinsic background of each individual probe, standardized 12 h dose rate measurements are performed in a lead castle and the intrinsic background is derived by subtraction of the residual component of 27 min^−1^ with a statistical uncertainty of the order of 0.3 min^−1^ (1 sigma).

**Figure 1.**
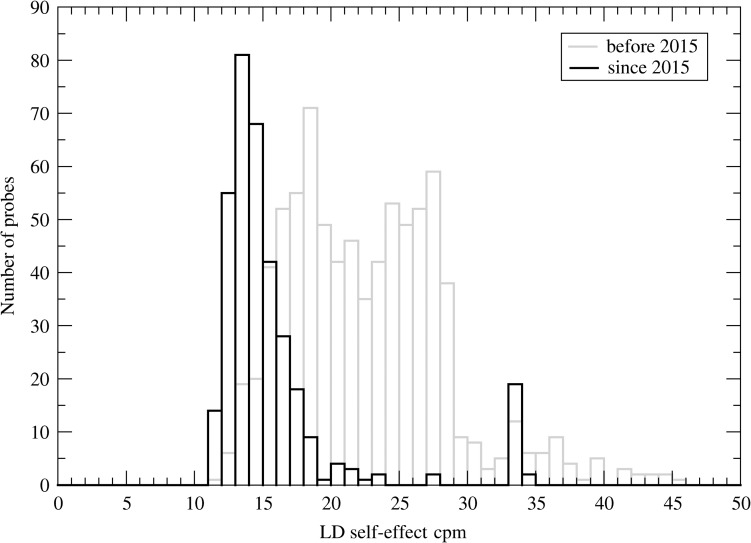
Frequency distribution of the intrinsic background of 1150 LD tubes. The data of LD tubes purchased until 2015 are shown in gray and that of tubes purchased since 2015 in black.

1150 probes have been characterized in the past 7 years. Figure 1 shows the frequency distribution of the intrinsic background of the LD tubes obtained differentiated between tubes purchased before (in gray) and after 2015 (in black). A large variability of the intrinsic background from 10 to 50 nSv h^−1^ is observed but it becomes obvious that over 15 years the mean intrinsic background is considerably reduced to 16 ± 5 min^−1^ with some outliers at ~35 min^−1^. The intrinsic background of HD tubes is 0.2 ± 0.02 min^−1^.

Since the intrinsic background of the LD and HD tubes is determined individually, the total uncertainty of one single background measurement is estimated as 10%. The variation of the SCR is negligible.

#### Calibration procedures

Calibration factors are determined for each probe individually at the irradiation facility of the Helmholtz center in Munich. This SSDL facility^(4)^ allows irradiations with seven ^137^Cs sources up to ~2 Sv h^−1^ and with five Co-60 sources up to 10 mSv h^−1^. The mean background dose rate level inside the irradiation facility indicated by GM tubes is 0.06 μSv h^−1^. For each individual detector, standardized calibration procedures are performed using three different ^137^Cs sources in a distance of 4 m with corresponding dose rate levels of 10 μSv h^−1^, 1 mSv h^−1^ and 100 mSv h^−1^. For each calibration level, ten 1 min measurements of both GM tubes are performed and compared with the 10 min background measurements before and after the irradiation. This procedure enables the calculation of calibration factors for both GM tubes individually.

**Figure 2.**
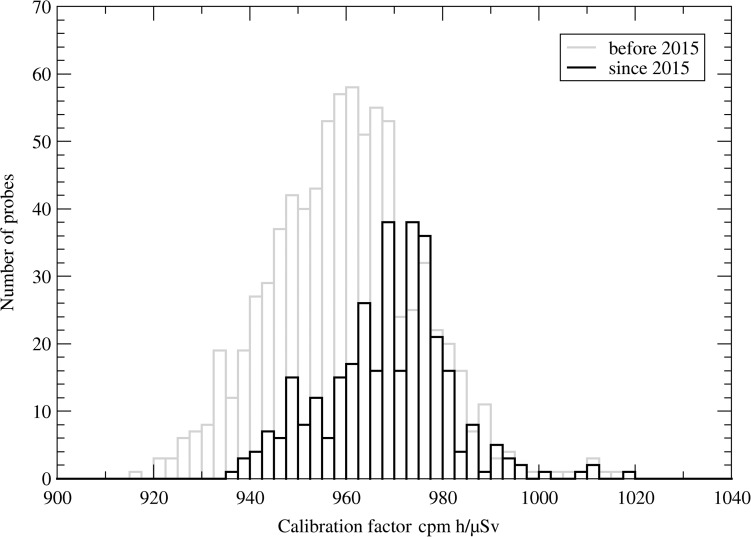
Frequency distribution of the calibration factors of 1150 LD tubes.

Figure 2 shows that the mean calibration factor of tubes manufactured before 2015 (in gray) and after 2015 (in black) changed from 960 to 968 ± 13 min^−1^ h μSv^−1^. Typical calibration factors of HD tubes are 1 min^−1^ h μSv^−1^.

Using the irradiation facility, non-linear effects of the relation between dose and count rate in the upper dose rate region were investigated. For different probe types, dead time coefficients were determined. Typically, the dead time of the LD and HD tubes used for probe types GS07 and GS08 is 3 × 10^−6^ min and 3 × 10^−7^ min, respectively. But non-linear effects have to be considered above 20 and 100 000 μSv h^−1^, respectively.

#### Temperature dependence of the probe

A climate chamber is used to investigate the temperature dependence of the indicated dose rate of the probes at a background level of ~0.06 μSv h^−1^. A standardized test procedure records counting rates of both GM tubes in a detector, in a controlled 48 h measuring cycle. In one cycle, the temperature starts at 20°C, reaches 55 and −20°C levels and returns to 20°C. For each level, readings are recorded for 6 h and the temperature drift of each probe is determined individually.

First, this procedure is used as a quality assurance check of the GM tubes. Only LD tubes not exceeding the count rate limit of 7 min^−1^ between −20 and 55°C are accepted for the use within the network. For the HD tubes the acceptance limit is 0.6 min^−1^ over the whole temperature range. Secondly, for probes which have passed this quality check, the uncertainty of the dose rate due to the variability of the temperature is assessed.

#### Photon energy response

The energy response in terms of H*(10) of the LD and HD tubes was determined in different EURADOS inter-comparison experiments^(7)^ using mono-energetic gamma sources. Between 60 and 660 keV, the deviation of the response from a flat curve is below 10% for normal radiation incidence. Between 60 and 1300 keV, the deviation is below 25%.

Real environmental spectra have a broad energy distribution. They show a broad continuous Compton background and emission lines of naturally occurring radionuclides, e.g. ^40^K, ^214^Bi, ^214^Pb, ^228^Ac, ^212^Pb, ^208^Tl and artificial long-lived radionuclides (^137^Cs) from global fallout or accidents like Chernobyl.

#### Determination of the secondary cosmic component

The secondary cosmic component (SCR) consists mainly of muons, but also of electrons, positrons, neutrons and gamma rays. The SCR is generated by interactions of the primary cosmic radiation (mainly protons) with atoms of the upper atmosphere^(8)^. Different types of detectors respond differently to SCR. GM and proportional counters overestimate the SCR (in our case of GM tubes by a factor of 1.3), in contrast to scintillation counters, which often underestimate the SCR. The energy spectrum and measured dose rate of the SCR depends on the altitude above sea level, the geographical longitude and the actual air pressure.

For the GM probes used by BfS, the altitude dependence due to SCR in Germany has been determined experimentally^(9)^. An approximation to these results is the following formula for the dose rate of the SCR:

(1) $${\mathrm{dH}}^{*}{}_{\mathrm{SCR},\mathrm{GM}}\left(10\right)/\mathrm{dt}=0.0421\;*\;\exp\left(0.0003\;*\;a\right)\;*\;\mathrm{Sv}\;h^{-1}$$

with a: height above sea level in meters.

Based on this formula, the ADER reading of the GM tubes due to SCR is calculated for each individual station. Comparing different approaches, a detailed discussion of this topic was performed by Bossew *et al.*^(10)^ leading to an uncertainty of ~15%.

Air pressure sensors are installed in new ADER probe types GS07 and GS08. They allow the calculation of the barometric altitude and reduce the corresponding uncertainty of the SCR component to ~5%.

In the future, BfS network will combine GM based probes with modern probe types based on LaBr_3_ detectors, which underestimate the SCR component. Absolute readings of different probe types can be compared, if the under-response of spectrometers to SCR is taken into account.

#### Quality assurance and quality control procedures

Quality assurance and quality control (QA/QC) procedures focusing on detector calibration and electrical safety tests are planned to be carried out at monitoring stations once within a fixed period. In addition, gamma spectrometric *in-situ* measurements using portable hyper-pure Germanium detectors (HPGe) are taken and the site is characterized to keep track of changes of site conditions. Electrical safety tests have to be performed every 4 years and batteries are exchanged every 8 years. These tests also allow the optimization of the overall electronic performance of the technical equipment of the ADER network, since a proper electrical installation also leads to an improved stability with respect to electronic noise.

To test the whole detector system including data transmission from the probe to the data logger and to the central servers at increased count rates a periodic quality assurance test is performed every 3 years at each site using a ^137^Cs source. These tests are complemented by the daily check during the data validation process in the services nodes by specialized experts. Each anomaly in the measurement system including data transfer is noticed and failure analysis is performed. These daily quality checks are supported by specialized tools to control the long-term performance of LD and HD tubes.

#### Initial and periodic performance tests with ^137^Cs source

To ensure a high quality functionality and to uncover possible failures or warranty claims, initial performance tests of newly delivered or repaired probes are taken at the maintenance center in Neuherberg for all probes. Probes are irradiated at dose rate levels at ~10, 1000 and 100 000 μSv/h and observed dose rate are compared with reference values. Using ^137^Cs sources below permitted limit, periodic quality assurance tests of each probe are performed after installation at the measuring site. Dose rate levels of ~5 μSv/h were achieved and obtained values are compared with reference values. These tests allow the inspection of the complete installation without taking the measuring system out of service for an extended period. In these tests, the long-term responses of the probes are compared with their initial parameters. The test procedure has been started in April 2001. The 98% of the measuring systems passed the criteria of these tests. The error rate is constantly falling. In cases of errors, the causes were mainly specific effects in the tubes.

**Figure 3.**
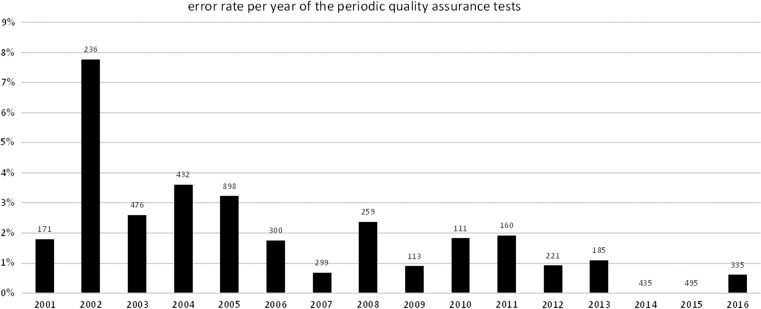
Annual number of periodic probe quality assurance tests and corresponding failure rate.

Figure 3 shows the annual failure rate of the actual performed periodic tests between 2001 and 2016. In 2014 and 2015, 435 and 495 tests were performed, respectively, but no failures were detected. Due to changing operation conditions between 2001 and 2016, the required period of 3 years is not always met. The failure rate has been below 1% in the past 5 years since 2012.

#### Electrical safety tests of ADER monitoring stations

Defects in electrical systems and equipment go along with an increased risk of accidents and fire. Together with other maintenance tasks, electrical specialists regularly check the safety of the measuring systems, at least every 4 years. Electrical safety tests according to the German Statutory Accident Insurance (DGUV) regulation, part 3 and DIN VDE/EN standards are required by law in Germany. Failures are removed immediately. These tests improve the electrical safety of the measuring systems and also reduce electromagnetic interferences, thus leading to an improvement of the overall performance of the station.

**Figure 4.**
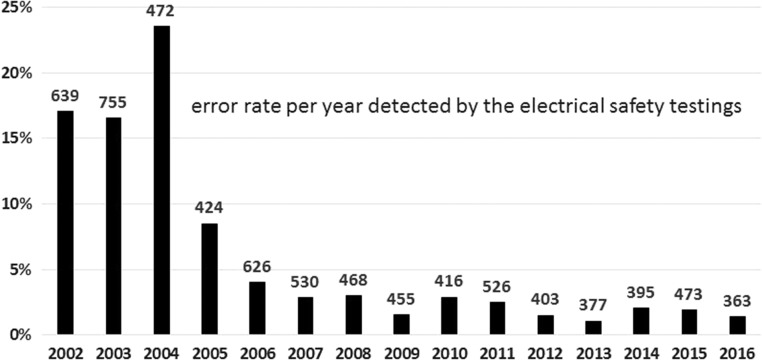
Annual number of performed electrical safety tests and observed annual failure rate.

Figure 4 shows the annual failure rate of electrical safety tests between 2002 and 2016. For more than 5 years ago, the annual rate of electrical failures has been below 1%. Approximately 400 tests are performed per year.

## DATA PROCESSING AND ANALYSIS

### Central server nodes

As part of the redundancy concept, the network is operated from six network centers located in Berlin, Bonn, Freiburg, Munich, Salzgitter and Rendsburg. Each center is fully capable of polling and processing all network data. In routine mode, data are retrieved from all stations every 6 h. These data are immediately presented as hourly mean values to the public without manual data validation (http://odlinfo.bfs.de). In routine mode, data are manually validated once a day. Data which do not pass this manual check are marked and removed from the web-site during the next update interval. The polling frequency is changed to 10 min in an emergency case, allowing almost real-time mapping of the radiological situation.

### Data treatment in the data logger

The data logger is typically installed in a building close to the detector and receives counts from both GM tubes, the echo counts from the LD tube, temperature and humidity data and a flag indicating a correct high voltage for the tubes via serial RS485 interface every 10 s in polling mode.

#### Outlier detection

Six 10 s readings of counts are added to a 1 min (60 s) sample. The 10 s interval was selected to enable the detection of outliers due to electronic noise or nearby lightning strikes, which evoke a massive amount of pulses in a very short time. These high readings in a single 10 s interval will result in a high standard deviation of the sample. As a consequence, this dataset will be flagged in the database for manual inspection.

#### Alerting mechanism of the data logger

Based on the 1 min values, the mean and the standard derivation are calculated every minute using a peak elimination algorithm. This algorithm is a recursive procedure based in the first step on a preliminary calculation of the mean and the standard deviation from all readings typically in a 7-day interval. In the subsequent steps, all peaks in the time series of the last 1000 1 min values (representing a period of ~17 h), which deviate by more than two sigma from the mean are marked. This procedure is repeated recursively only with the values which are not marked until no further peaks are identified. Then, the peak eliminated mean and standard deviation are used to dynamically calculate the alerting threshold of the station according to the following formula:

(2) Alertingthreshold=mean+standarddeviation∗6.0

This dynamical threshold has the advantage that systematic changes at the station are taken into account (e.g. snow shielding of the terrestrial component). If the actual readings from the probe are higher than this alerting threshold for more than 3 min, spontaneous data transfer is initiated by the data logger. The counts from the probe, echo counts, temperature and humidity data, the high voltage flag, technical status as well as the mean and the standard deviation are transmitted to the central database for further analysis. The station stays in a 10 min transfer mode until the values are below the threshold again, but at least for 2 h. The same mechanism is applied in case of malfunctions detected by the data logger (e.g. battery failure or external power blackout).

#### Early warning mechanism

When two neighboring stations (distance <30 km) detect increased radiation levels within 1 h, the officer on duty is notified via cell phone. He has to analyze the radiological situation within 1 h^(11)^. Assuming a typical ADER of 0.08 μSv h^−1^, the alerting level is ~0.11 μSv h^−1^.

**Figure 5.**
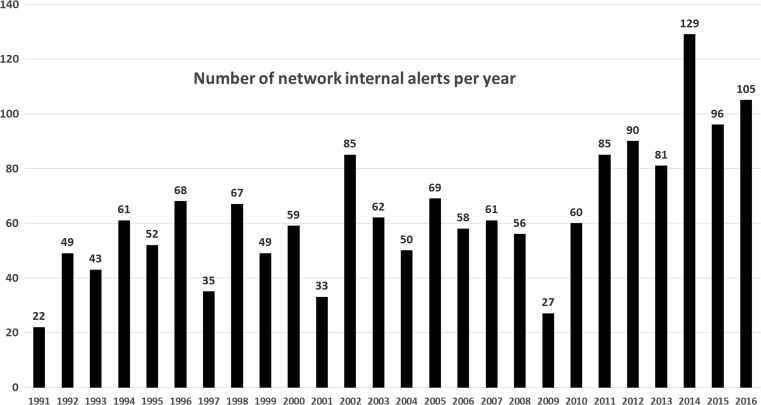
Annual number of network internal alerts from 1991 until 2016.

In Figure 5, the number of internal network alerts from 1991 to 2016 is shown. Since 2006 data loggers have successively been replaced by new Linux based systems with dynamic alerting threshold. In old data loggers the alerting levels were based on station specific, fixed parameters.

### Dose rate calculation

One of the basic principles of the data processing concept implemented by BfS is to query only raw data from the probes and to calculate the ADER based on intrinsic background and calibration parameters remotely. This avoids update mechanisms for calibration and alerting parameters on each data logger.

The ADER is calculated from raw count rates of the LD and HD tubes separately. In the next step the dose rate is compared with the limits of the measurement ranges of both GM tubes. In case that the dose rate is below the HD range (approximately below 100 μSv h^−1^), the dose rate from the LD tube is used. In case the readings are within the range of overlap, the mean of the HD and LD dose rate is calculated. Above the overlap range, the dose rate from the HD tube is used.

### Data validation

All dose rate data are compared with upper and lower validation thresholds. The upper validation threshold is the sum of median and variance observed in the proceeding time frame of 7 days. The lower validation threshold is median minus variance. Data outside the validation thresholds are automatically flagged for review once a day in routine operation.

**Figure 6.**
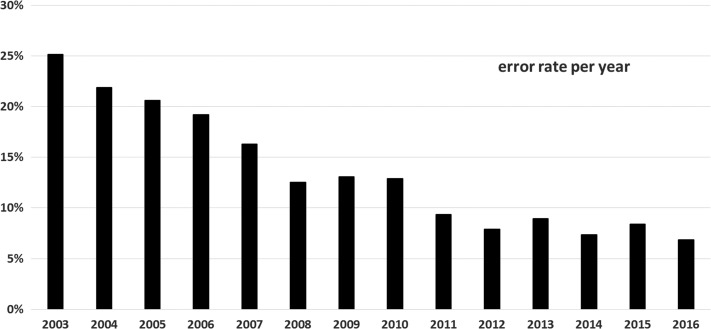
Percentage of stations with at least one invalid ADER reading per year.

The percentage of stations with at least one invalid reading per year is shown in Figure 6. This number also includes malfunctions due to on-site maintenance work and other activities at the station.

## CONTRIBUTIONS TO THE AMBIENT DOSE EQUIVALENT RATE

Over longer periods in time the measurement results of the environmental ADER at fixed position shows a considerable variability. The following natural contributions are present (the given number quantifies the effect, which may occur under typical conditions):

- Snow coverage: leading in some cases to the complete shielding of the terrestrial component
- Soil humidity: <0.01 μSv h^−1^ decrease
- Radon concentrations in air: <0.01 μSv h^−1^ increase
- Short term precipitation effects: <0.15 μSv h^−1^ increase

### Radon concentrations in air

In Germany the mean outdoor radon concentrations are below 30 Bq m^−3^ with an arithmetic mean of 10 Bq m^−3^ and a standard deviation of 5 Bq m^−3^. Using the conversion factor of 0.5 nSv h^−1^ Bq^−1^ m^3^ reported by Bossew *et al.*^(10)^ and assuming an equilibrium factor of 0.5, the activity concentration of 10 Bq m^−3^ leads to a dose rate of 2.5 nSv h^−1^. The contribution of radon to the ADER is strongly increased by inversion layers.

### Precipitation events and soil humidity

The ADER can increase up to 0.15 μSv h^−1^ in case of a precipitation event, since natural radon progeny are deposited on the ground. It is dominated by the Rn-222 progeny Bi-214 and Pb-214 with half-lives of 20 and 30 min, respectively. After the rain the ADER drops down to a value of 0.01 μSv h^−1^ below the baseline. The water content of the soil increases and additionally shields the terrestrial ADER.

These effects have been investigated in detail in the framework of a master thesis at the University of Basel, Switzerland, to develop procedures and to describe the effect of the correlation between ADER and soil moisture experimentally^(12,13)^.

### Cesium background

**Figure 7.**
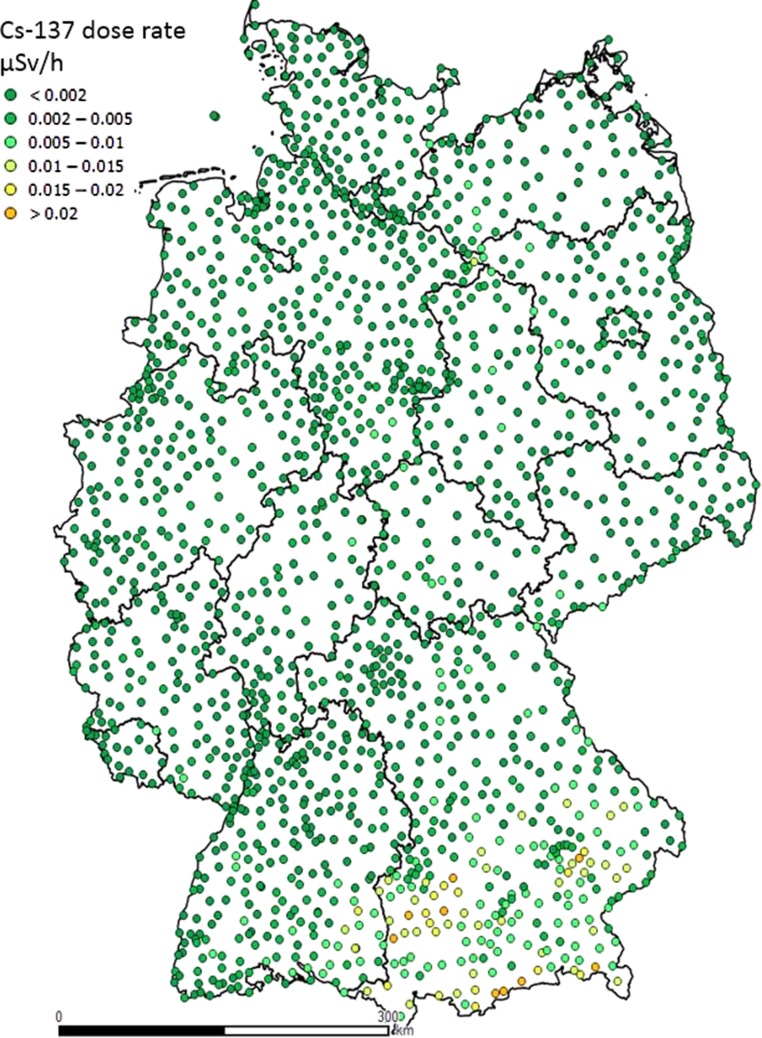
Dose rate contribution of 137Cs in Germany assessed from measured spectra between 2012 and 2016 using HPGe detectors at BfS monitoring network sites.

BfS operates six mobile gamma spectrometric measuring systems equipped with high purity germanium detectors (HPGe detectors), which are used to perform nuclide specific measurements at each site of the stationary network every 3 years. Figure 7 shows the calculated nuclide specific dose rate contribution of ^137^Cs in the years from 2012 to 2016.^(14)^ Typically the nuclide specific dose rate is below 0.002 μSv h^−1^. Only at two sites in areas affected by the reactor accident in Chernobyl 1986, maximum values up to 0.014 μSv h^−1^ are observed.

### The terrestrial component

Main contributions of the terrestrial radiation come from primordial radionuclides ^238^U, ^232^Th and ^40^K and their decay products. A detailed study on the correlation between ADER and the geology based on the gamma spectrometric *in-situ* measurements by BfS was reported in a diploma thesis at the University of Hanover^(15)^.

Neglecting contribution from radon progeny in air, the terrestrial component is calculated simply by subtracting the SCR component from the gross ADER.

(3) $${\mathrm{dH}}^{*}{}_{\mathrm{TR}}\left(10\right)/\mathrm{dt}={\mathrm{dH}}^{*}\left(10\right)/\mathrm{dt}-{\mathrm{dH}}^{*}{}_{\mathrm{SCR}}\left(10\right)/\mathrm{dt}$$

**Figure 8.**
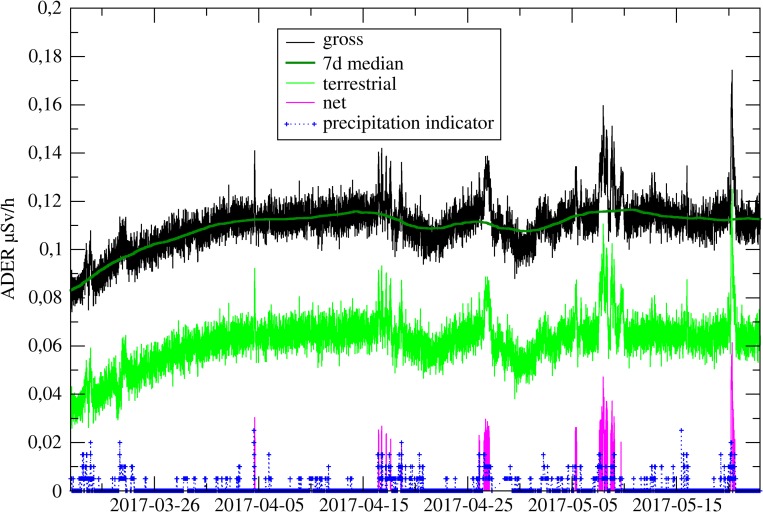
Time series of gross ADER, 7 days running median of gross ADER and terrestrial contribution from monitoring station at INTERCAL on mount Schauinsland close to Freiburg. Net ADER values above 0.02 μSv h^−1^ and an indicator for precipitation (arbitrary units) derived from weather radar data are given.

In Figure 8, the time series of the response to the SCR, the gross ADER and terrestrial component are shown.

### Net ADER

To account for long-term variations of the ADER, the median method is used. From the gross ADER data the sliding 7 days median is calculated. The subtraction of the 7 days sliding mean from the gross ADER leads to the net ADER. Typically, the net ADER follows a smooth base line with statistical fluctuation. Mainly due to precipitation events, the net ADER shows short elevated levels.

As can be seen from Figure 8, the 7-day median smoothly follows the base line of the 10 min gross ADER. Increased dose rate readings with maxima up to 0.1 μSv h^−1^ are visible on top of this baseline, which are caused by the wash-out of radon progeny during precipitation periods. These so-called ‘rain events’ typically have a duration of several hours up to 1 day. They have a short rise time and a mean decay time of 30 min for short spot rain events, which corresponds to the half-life of the relevant radon progeny. Figure 8 shows also an increase in the baseline in the summer months, which is caused by a reduced water content of the soil at this station.

An indicator for precipitation is also included in this figure (in arbitrary units). Weather radar data in 5 so-called reflectivity levels are provided by the German Weather Service (DWD) for whole Germany. Precipitation information is only used to support the data validation process performed by the officer on duty and not included in the automatic data analysis process.

Using the net ADER, contributions from detector sensitivity, self-effect and SCR drop out. When using this method, only enhanced net ADER above the statistical fluctuations become visible, which are caused by rain effects.

## MEASUREMENT UNCERTAINTIES

### Uncertainty budget

For typical background level in Germany (0.08 μSv h^−1^), the expected counting rate of the LD tube is ~100 min^−1^ and the counting rate distribution is Gaussian. The standard deviation is 3 min^−1^ (10 min interval) respectively 1.3 min^−1^ (1 h interval). Thus, the 2*σ* uncertainty due to statistical fluctuations is ~6% for 10 min intervals and 3% for 1 h intervals.

The temperature dependence on probe readings is typically well below 5 nSv h^−1^ and the uncertainty of calibration factors is <10%. Using this information, the typical 2*σ* uncertainty of ADER readings from BfS probes is <30%.

### Site characterization

Observed dose rate from a specific site is influenced by the location of the probe. The German ADER monitoring network use an ‘ideal site’ approach: The probes are installed 1 m above on an extended flat and smooth grassland. Obviously, buildings and walls in the vicinity of a probe will influence the measured dose rate by shielding effects especially in case of freshly deposited activity after an accidental release. Other relevant disturbing objects are trees, water surfaces and sealed surfaces^(16)^. A site characterization technique was developed to discuss these influence of probe location for real sites within the German monitoring network. In the framework of the MetroERM project, a simple site characterization method was developed^(17)^. This method is useful to discuss the impact of probe location on measured data. Data assimilation techniques used by decision support systems like RODOS will profit from this approach.

#### Installation of detectors on walls

**Figure 9.**
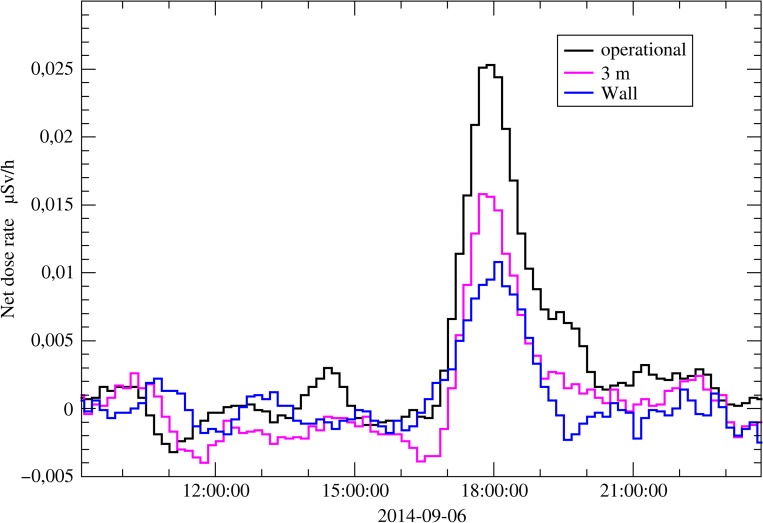
Comparison of net ADER readings of three stations installed in Neuherberg during a precipitation event. The data in black are from a detector installed at the free field, the data in magenta from a detector mounted in a distance of 3 m away from a building and data in blue from a detector directly in front of the wall of the building.

At the INTERCAL facility on mount Schauinsland near Freiburg^(5, 6)^, one additional probe was installed directly fixed on the wall of a building. In addition, one other detector was installed 1 m above ground in a small forest close to the INTERCAL facility. At the Neuherberg reference site, one probe was installed 1 m above ground on the wall of a building and another probe was installed 1 m above ground at a distance of 3 m from the same building. At both reference sites, observed dose rates during rain events were compared. At the INTERCAL facility, it was observed that the net dose rate was smaller by a factor of 2 during rain events at both non-standard locations. At Neuherberg, the same reduction factor of net dose rates was observed during rain events for the probe fixed on the wall and a factor of 1.5 was found for the probe installed 3 m away from the building (Figure 9).

#### Installation of detectors at different heights

**Figure 10.**
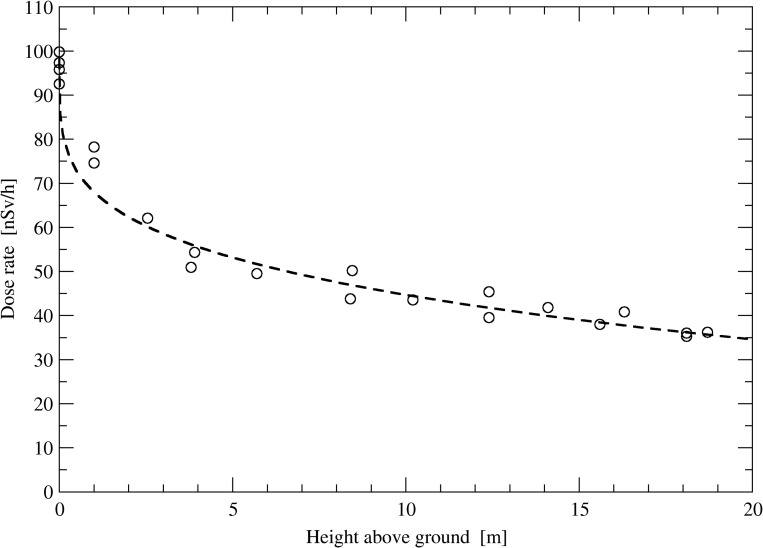
Dependence of the gross ADER on the altitude above ground obtained at a look-out tower at mount Schauinsland.

Observed dose rate data depend on the height of the fixed probe above ground. To show this influence, an experiment was performed on a wooden look-out tower at mountain Schauinsland^(18)^. The tower has a height of about 18 m with five platforms. Dose rate was measured at different heights of the look-out tower. One probe was placed 1 m above ground level to follow varying fluctuations (e.g. due to changing meteorological conditions). Exemplarily, Figure 10 shows the influence of the probe height above ground on observed dose rate.

## SUMMARY AND CONCLUSION

The data exchange platform EURDEP collects and provides ADER data from all European early warning networks in almost real-time on behalf of the European Commission. Today, ADER data are collected from 30 European countries, using a considerable number of different detector types and following different national policies. The comparability of these data is crucial for a meaningful interpretation, not only in the event of a nuclear accident with trans-boundary implications, but also for the correct interpretation of the data under natural background conditions. Furthermore, data assimilation techniques used in decision support systems like RODOS^(19)^ strongly depend on harmonized data complemented by an appropriate uncertainty budget based on both, the physical characteristics of the detectors and on an appropriate description of effects from the measurement site. Therefore, metrological sound ambient dose equivalent rate data are a prerequisite for adequate environmental radiation monitoring in Europe.

Data harmonization of dose rate data should be based on mechanisms to decompose the ambient dose equivalent rate taking into account the intrinsic background and the response of the detector to secondary cosmic radiation. In this way, the terrestrial gamma dose rate can be calculated, which may include, e.g. minor fractions caused by ^137^Cs from the Chernobyl fallout and air-borne activity due to radon progeny and is influenced by soil moisture and possible snow cover in winter.

The operation of a network following the design rules and procedures to retrieve harmonized data requires special efforts. Necessary steps were described here to obtain the terrestrial dose rate from the raw readings of a detector. So far, many ADER monitoring networks in Europe have not applied harmonization procedures, because additional efforts are required. In addition, the AIRDOS database in the EURDEP system requires updates of the parameters of all ~4700 ADER monitoring stations operated in EURDEP on a regular basis. Appropriate interfaces to allow the direct update of such data are necessary and need to be agreed and developed.

The calculation of the net ADER from all stations in EURDEP could be a first step in the harmonization process. In addition, the proposed site characterization procedure^(17)^ should be used. In case of an accident, maps of net ADER calculated from EURDEP data would help to better identify affected area in Europe.

Facing all efforts and different approaches in the last 15 years aiming at the harmonization of dose rate data in Europe, one can conclude that much more time and further profound efforts are needed to reach the goal, that metrological harmonized data can be retrieved from the EURDEP database including uncertainty information.
